# Calcium Dependence of Eugenol Tolerance and Toxicity in *Saccharomyces cerevisiae*

**DOI:** 10.1371/journal.pone.0102712

**Published:** 2014-07-18

**Authors:** Stephen K. Roberts, Martin McAinsh, Hanna Cantopher, Sean Sandison

**Affiliations:** 1 Division of Biomedical and Life Sciences, Faculty of Health and Medicine, Lancaster University, Lancaster, United Kingdom; 2 Lancaster Environment Centre, Lancaster University, Lancaster, United Kingdom; Louisiana State University Health Sciences Center, United States of America

## Abstract

Eugenol is a plant-derived phenolic compound which has recognised therapeutical potential as an antifungal agent. However little is known of either its fungicidal activity or the mechanisms employed by fungi to tolerate eugenol toxicity. A better exploitation of eugenol as a therapeutic agent will therefore depend on addressing this knowledge gap. Eugenol initiates increases in cytosolic Ca^2+^ in *Saccharomyces cerevisiae* which is partly dependent on the plasma membrane calcium channel, Cch1p. However, it is unclear whether a toxic cytosolic Ca^2+^elevation mediates the fungicidal activity of eugenol. In the present study, no significant difference in yeast survival was observed following transient eugenol treatment in the presence or absence of extracellular Ca^2+^. Furthermore, using yeast expressing apoaequorin to report cytosolic Ca^2+^ and a range of eugenol derivatives, antifungal activity did not appear to be coupled to Ca^2+^ influx or cytosolic Ca^2+^ elevation. Taken together, these results suggest that eugenol toxicity is not dependent on a toxic influx of Ca^2+^. In contrast, careful control of extracellular Ca^2+^ (using EGTA or BAPTA) revealed that tolerance of yeast to eugenol depended on Ca^2+^ influx via Cch1p. These findings expose significant differences between the antifungal activity of eugenol and that of azoles, amiodarone and carvacrol. This study highlights the potential to use eugenol in combination with other antifungal agents that exhibit differing modes of action as antifungal agents to combat drug resistant infections.

## Introduction

There is increasing interest in the use of plant-derived antimicrobial compounds as both natural preservatives and in the treatment of fungal infections. A driver for this interest is that antifungal therapies are limited to a few classes of compounds which are undermined by the emergence of resistance and tolerance coupled with innate toxicity of these compounds to the host organism [1].

Eugenol is the major constituent of essential oils from clove, cinnamon and bay leaves and is a member of a wider group of diverse amphipathic phenolic compounds which display antifungal activity [2]. Despite the therapeutic potential of these compounds, relatively little is known about their mode of action and the mechanisms employed by fungi to resist their toxicity. Several modes of action have been proposed including disruption of ion homeostasis [3], nonspecific lesion of the plasma membrane (resulting in leakage of cell contents; [4]), disruption of amino acid metabolism [5] and generation of oxidative stress [6]. However, the mechanism of killing remains to be fully elucidated and consequently we know little of the defence mechanisms employed by fungi to resist the toxic effects of eugenol (and related amphipathic phenolic compounds). Such knowledge will be essential in facilitating the application of these promising therapeutic compounds.

Previous studies have shown that amiodarone and carvacrol, two amphipathic phenolic compounds related to eugenol, induce increases in cytosolic Ca^2+^ in *Saccharomyces cerevisiae* and that their toxicity correlates with the amplitude and duration of cytosolic Ca^2+^ elevation; consequently, it has been proposed that the Ca^2+^ elevation represents a toxic shock responsible, at least in part, for the antifungal activity of these compounds [7], [8], [3]. Recently however, Roberts et al. [9] have shown that although eugenol also induces a cytosolic Ca^2+^ elevation in yeast the *cch1Δ* mutant (which is deficient in a subunit of the yeast high affinity plasma membrane Ca^2+^ channel) exhibits reduced Ca^2+^ influx in response to eugenol but is hypersensitive to eugenol. This raises the exciting possibility that the mechanisms by which yeast respond to eugenol and the related compounds amiodarone and carvacrol differ and that a Cch1p-mediated Ca^2+^ influx forms part of a cell signalling response to enable yeast to survive eugenol stress.

The present study investigates the role of Ca^2+^ in eugenol toxicity in detail. We show that the eugenol-induced cytosolic Ca^2+^ elevation in yeast is unlikely to represent a toxic burst of Ca^2+^. Importantly, Ca^2+^ influx appears to be limited to a signalling role that is crucial for protecting yeast against eugenol stress.

## Materials and Methods

### Strains, media and reagents

Single and double *mid1Δ* and *cch1Δ* mutants were derived from the parental *S. cerevisiae* strain JK9-3da (Mata, leu2-3, 112*Δ*, his4*Δ*, trp1*Δ*, ura 3-52*Δ*, rme1, HMLa) by replacing the MID1 and CCH1 genes by a KanMX cassette [10]. Yeast strains were transformed with pEVP11/AEQ (a plasmid bearing apoaequorin gene under constitutively expression and a LEU2 marker, generously provided by Dr Patrick Masson, University of Wisconsin-Madison, Wisconsin, US) as previously described [11]. Unless otherwise stated, yeast strains were cultured at 30°C in standard synthetic complete media minus the addition of leucine (SCM-leu; Foremedium, UK). SCM-leu-Ca is synthetic minimal glucose media [12] modified to contain no added calcium by replacing the calcium pantothenate with sodium pantothenate and omitting the addition of CaCl_2_ [10] and supplemented with complete supplement mixture-leu (Foremedium, UK).

*vcx1Δ* and *pmc1Δ* mutants were derived from the parental strain *S. cerevisiae* strain BY4742 (Matα, his3*Δ*1, leu2*Δ*0, lys2*Δ*0, ura3*Δ*) by gene replacement with a KanMX cassette (EUROSCARF) and cultured in YPD contained 1% yeast extract and 2% peptone. All growth media contained 2% (w/v) glucose (and 2% (w/v) agar for solid media), and where indicated, supplemented with BAPTA (1,2-bis(o-aminophenoxy)ethane-N,N,N′,N′-tetraacetic acid), EGTA (ethylene glycol tetraacetic acid) or CaCl_2_ using stock solutions of BAPTA (10 mM BAPTA, 10 mM HEPES, 2% glucose, pH 7.5 with Tris base), EGTA (0.5 M EGTA, 10 mM HEPES, 2% glucose, pH 7.0 with NaOH) or 2 M CaCl_2_. pH was approximately 6.5 for YPD-based growth media and between 5.0 and 5.5 for SCM-based growth media. Eugenol, isoeugenol, estragole, o-eugenol, acetyl eugenol and methyl eugenol (Sigma Aldrich) were in liquid form and made to 100x stocks in ethanol and stored at 4°C.

### Luminometry

Cells expressing apoaequorin were grown overnight in SCM-leu in a shaking (150 rpm) incubator to an optical density at 600 nm (OD600) of 8 (approximately 1×10^8^ cells/ml). OD600 was determined after 8x dilution of culture in water. To obtain cells in mid-log growth phase, 0.5 ml of overnight cultures were subcultured into 10 ml of fresh SCM-leu to give an OD600 of 0.8 and incubated shaking at 150 rpm for up to 3 hours until an OD600 between 1.6 and 2.4 was reached. Cells were pelleted (at room temperature using 200 g for 5 minutes) and resuspended in fresh SCM-leu to an OD600 of 2.4. 4 µl of 0.5 mM coelentrazine (Prolume, USA) in absolute methanol was added to 1 ml of cells and incubated in the dark for 2 hours at 30 C shaking at 150 rpm. Coelentrazine loaded cells were pelleted in a microcentrifuge (5000 rpm for 20 seconds) and resuspended in fresh SCM-leu to an OD600 of 8. Luminescence from 20 µl samples of mid-log growth phase cells was recorded as previously reported [9]. Eugenol (and eugenol derivatives) were added to samples at indicated concentrations (containing 1% ethanol) in either 200 µl of Ca buffer (10 mM CaCl_2_, 10 mM HEPES, 2% glucose, pH 7.5 with Tris base), BAPTA buffer (10 mM BAPTA, 10 mM HEPES, 2% glucose, pH 7.5 with Tris base) or SCM-leu. Luminescence (expressed in arbitrary units (AU) per 0.2 seconds) was measured for up to 10 minutes after which cells were lysed with 1.6 M CaCl_2_ in 20% (v/v) ethanol to determine total (summed) luminescence. Total luminescence was in much greater excess over luminescence induced by eugenol (or eugenol derivatives) indicating that the availability of aequorin-coelentrazine complex was sufficient for the reporting induced Ca^2+^_cyt_ elevations.

### Toxicity assays

#### Transient exposure to eugenol – dot drop assays

1 ml of overnight cultures of JK9-3da (and derived mutants) was added to 9 ml of fresh SCM-leu to an OD600 of 1.6 and incubated for approximately 4 hours shaking at 150 rpm until an OD600 between 3 and 4 was reached and cells were in mid-logarithmic growth phase. Culture was pelleted (200 g for 5 minutes) and resuspended in 5 ml of 2% glucose, repelleted and resuspended in 1 ml of 2% glucose. 0.5 ml cell samples were pelleted in a microcentrifuge (5000 rpm for 20 seconds) and resuspended in 0.5 ml of Ca buffer or BAPTA buffer and adjusted to an OD600 of 20. 100 µl of cells were pelleted and resuspended in 0.5 ml of corresponding buffer containing eugenol and incubated at room temperature for 10 minutes (unless otherwise stated). Following incubation, cells were pelleted in a microcentrifuge and resuspended in 1 ml of SCM-leu, repelleted and resuspended in 100 µl of SCM-leu. 10-fold serial dilution was performed in sterile water and 5 µl of each dilution was placed onto SCM-leu containing 2% agar. Images of plates were taken after two days growth.

#### Persistent exposure to eugenol - dot drop assays

JK9-3da (and derived mutants) were cultured overnight in 10 ml SCM-leu to an OD600 of 8, pelleted (200 g for 5 minutes) and resuspended in 10 ml of sterile water and resuspended to 0.5×10^8^ cells/ml. Following 10-fold serial dilution of each yeast strain using sterile water, 5 µl drops were spotted onto YPD (1% yeast extract, 2% peptone, 2% glucose, 2% agar) plates containing varying concentrations of eugenol. Images of plates were taken after two days growth at 30 C.

#### Determination of IC_50_ values

Sensitivities of yeast growth to eugenol (and derivatives) were assayed by a dilution method in 24 well plates as described by Edlind et al. [13]. Briefly, overnight cultures in SCM-leu were pelleted and washed in 2% glucose, re-pelleted and diluted in either SCM-leu or SCM-leu-Ca supplemented with either Ca^2+^ or BAPTA to 10^−4^ cells/ml and 1 ml aliquoted to wells except the initial well in which 2 ml was aliquoted. Eugenol, eugenol derivatives or BAPTA were added to the first well and a two-fold dilution series achieved by mixing, removing 1 ml and adding this to the second well; this was repeated for subsequent wells except the final well which served as a eugenol (or BAPTA)-free control. Plates were sealed and incubated shaking at 150 rpm for 48 hours after which OD600 was determined (for OD values greater than 1, a 10x dilution of the sample was performed). For BY4742 strains, sensitivities to eugenol were determined as above except culture media was YPD. Student' *t* tests were performed on the IC_50_ values to determine p values and whether mean IC_50_ values were significantly differently.

## Results and Discussion

### Eugenol toxicity is not coupled to Ca^2+^ influx

**Figure 1:**
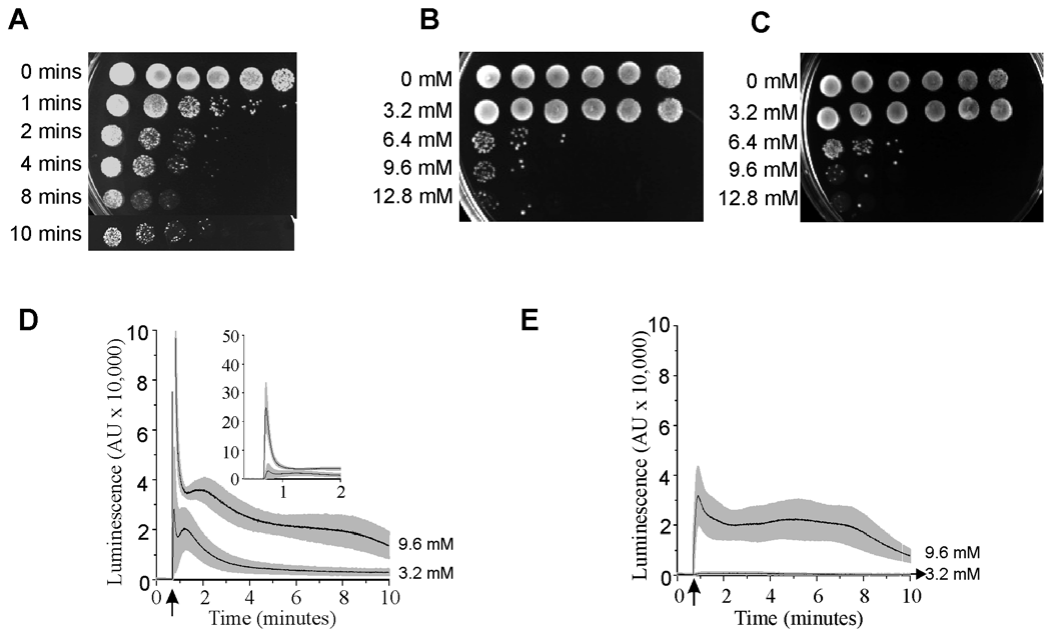
Eugenol toxicity is not dependent on Ca^2+^ influx. A) Time dependence of eugenol toxicity. Viability of *JK9-3da* cells after exposure to 6.4 mM eugenol suspended in Ca buffer for 1, 2, 4, 8 and 10 minutes. Yeast cultures are spotted on to SCM-leu media containing 2% agar; left most spots are growth after 2 days following inoculation with 5 µl of culture. Serial 10-fold dilution of the left most inoculum is shown to the right. B) as A except cells were exposed to varying concentrations of eugenol (as indicated) for 10 minutes in Ca buffer. C) as B except cells were exposed to eugenol in BAPTA buffer. D) Eugenol-induced cytosolic Ca^2+^ elevation in the presence of extracellular Ca^2+^ in mid log growth phase yeast cells. Ca^2+^-dependent aequorin luminescence from *Jk9-3da* cells in response to 3.2 and 9.6 mM eugenol in Ca buffer. Eugenol was added at 40 seconds (indicated by arrow). Traces represent mean (± SEM) from at least 5 independent experiments. SEM values are illustrated using grey shading. Luminescence was recorded every 0.2 seconds and is expressed in arbitrary units (AU). Inset is data from the main figure on an expanded y axis. E) As D except eugenol was in BAPTA buffer.

Yeast cells were transiently exposed to toxic levels of eugenol for varying times before transferring on to agar-containing growth media to monitor cell viability. Figure 1A shows that transient exposure to 6.4 mM eugenol was fungicidal and that this toxic effect was apparent within one minute of exposure to eugenol and showed increasing toxicity up to 10 minutes. Therefore, to investigate the role of Ca^2+^ in eugenol toxicity, 10 minute transient exposure of wild type (*Jk9-3da*) yeast to varying concentrations of eugenol were performed in the presence (10 mM CaCl_2_; Figure 1B) and absence (10 mM BAPTA; Figure 1 C) of extracellular Ca^2+^ before immediately transferring to growth media to monitor cell viability. In both cases transient exposure of up to 3.2 mM eugenol was tolerated by yeast, however, at concentrations greater than 3.2 mM fungicidal effects were apparent. Interestingly, removal of extracellular Ca^2+^ had no apparent effect on eugenol toxicity towards yeast suggesting that an influx of Ca^2+^ across the plasma membrane is unlikely to play a role in eugenol toxicity. To gain further sights into the role of Ca^2+^ in mediating eugenol toxicity in *S. cerevisiae*, cytosolic Ca^2+^ was also monitored in cells following exposure to eugenol (in identical conditions to that used to assess cell viability shown in Figures 1B and C) using the genetically encoded reporter, aequorin, reconstituted with its cofactor, coelentrazine. In the presence of extracellular Ca^2+^, eugenol-induced cytosolic Ca^2+^ elevations in wild type yeast were characterised by a large transient increase immediately following addition of eugenol followed by a prolonged Ca^2+^ elevation for up to 10 minutes (Figure 1 D). Increasing the concentration of eugenol increased the magnitude of the Ca^2+^ elevations (Figure 1 D) whilst removal of extracellular Ca^2+^ abolished the large transient elevation in cytosolic Ca^2+^ (and reduced total eugenol-induced cytosolic Ca^2+^ elevations; Figure 1E). However, there was no correlation between eugenol toxicity and either the amplitude and duration of the Ca^2+^ elevation or Ca^2+^ influx across the plasma membrane. Toxicity resulting from transient exposure of eugenol and Ca^2+^ elevations were also investigated in the yeast Ca^2+^ channel mutants cch1*Δ*, mid1*Δ* and cch1*Δ* mid1*Δ* (Figure S1). Interestingly, the *mid1Δ* mutant consistently exhibited greater tolerance to transient exposure of high concentrations compared to the wild type and *cch1Δ* mutant yeast although as in wild type yeast there was no correlation between eugenol toxicity and cytosolic Ca^2+^ elevation; the Ca^2+^ elevation in the *mid1Δ* mutant was equivalent to that observed in wild type yeast. It is also notable that the *cch1Δ* mutants exhibited equivalent tolerance to transient exposure of eugenol compared to wild type yeast despite the cytosolic Ca^2+^ elevation being consistently reduced in the *cch1Δ* yeast (Figure S1). Taken together, these results show that eugenol toxicity is unlikely to be mediated by a pancellular “toxic” elevation in cytosolic Ca^2+^.

At higher concentrations (e.g. 9.6 mM) the eugenol-induced cytosolic Ca^2+^ elevation consists of two distinct components: a rapid transient increase in cytosolic Ca^2+^ due to Ca^2+^ influx across the plasma membrane (Figure 1D) followed by a prolonged elevation of cytosolic Ca^2+^, which is independent of extracellular Ca^2+^ (i.e. present in BAPTA-containing buffer; Figure 1E) and thus must result from a release of Ca^2+^ from intracellular stores. The transient Ca^2+^ influx across the plasma membrane is independent of the presence of Cch1p and Mid1p (Figure S1) revealing that this Ca^2+^ influx across the plasma membrane is via an unspecified pathway; this could reflect non-specific disruption of the plasma membrane and general cell leakage [14], [4] or hitherto unidentified Ca^2+^ specific entry pathways [15], [16], [17].

**Figure 2:**
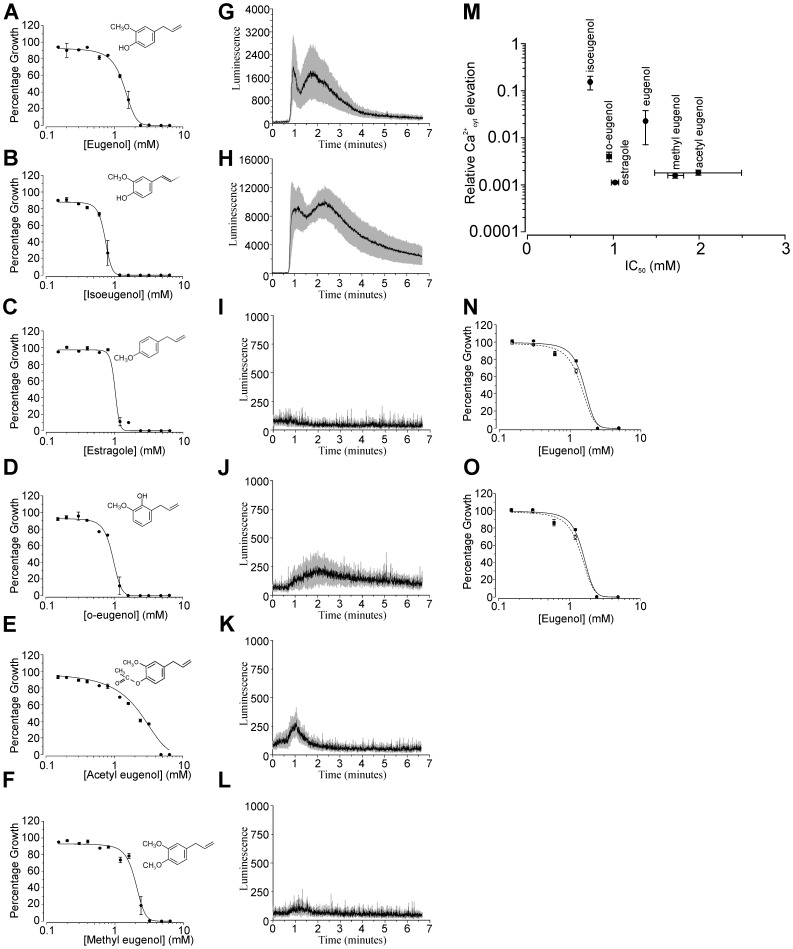
Eugenol toxicity is not dependent on Ca^2+^ influx. (A - F) Jk9-3da growth in SCM-leu containing varying concentrations of eugenol (A) or eugenol derivative (B–F). Absorbance was recorded after 48 hours incubation and is shown as growth (absorbance) relative to growth exhibited in eugenol or eugenol derivative-free control media (SCM-leu containing 1% ethanol). Data are fitted with the dose-response function min + (max-min)/1+ ((x/IC_50_)^−p^)) where p is the slope, IC_50_ is the eugenol concentration inhibiting 50% growth and min and max represent minimum and maximum relative absorbance values respectively. Mean values (± SEM) from 4 experiments are shown. (G–L) Ca^2+^-dependent aequorin luminescence from Jk9-3da cell in response to addition of 3.2 mM eugenol (G), isoeugenol (H), estragole (I), o-eugenol (J), acetyl eugenol (K) and methyl eugenol (L) added at 40 seconds in SCM-leu. Traces represent mean (± SEM) from at least 4 independent experiments. SEM values are illustrated using grey shading. (M) plot of IC_50_ values (from data shown in parts A–F) against Ca^2+^ elevations (determined from data shown in parts G–L). Relative Ca^2+^ elevations were calculated as the sum of luminescence resulting from addition of eugenol or eugenol derivative divided by total luminescence determined after lysis with 1.6 M CaCl_2_, 20% ethanol (see Materials and Methods). N–O) Growth of BY4742 (parental strain; solid symbols and line) and *pmc1Δ* (N) and *vcx1Δ* (O) yeast mutants (open symbols and dashed line) in YPD containing varying concentrations of eugenol. Growth was recorded as detailed in parts A–F. IC_50_ values are 1.56±0.189 mM (BY4742), 1.45±0.112 mM (*PMC1Δ*) and 1.41±0.083 mM (*VCX1Δ*).

To investigate the relationship between toxicity and cytosolic Ca^2+^ elevation further, we compared the toxicity of a range of eugenol derivatives with their ability to induce cytosolic Ca^2+^ elevations (Figure 2). Figure 2 M plots the concentration of eugenol (and derivatives) which results in 50% inhibition of yeast growth (IC_50_) against the cytosolic Ca^2+^ elevation induced following addition of 3.2 mM eugenol (or derivative). Notably, both estragole (hydroxyl group replaced with methoxy group) and o-eugenol (hydroxyl group moved to the carbon situated between the methoxy and allyl groups) exhibited significantly greater toxicity towards yeast (1.017±0.0435 mM and 0.949±0.0285 mM respectively) than eugenol (p<0.02 and <0.01 respectively) but with reduced elevation of cytosolic Ca^2+^. Isoeugenol however (which differs from eugenol in the position of the double bond in the allylic side chain) was significantly more toxic to yeast than eugenol (IC_50_ was 0.73±0.0096 mM compared to 1.375±0.0349 mM for eugenol; p<0.01) and induced greater cytosolic Ca^2+^ elevation compared to that for eugenol. Taken together, these results show no correlation between Ca^2+^ elevation and toxicity and do not support a mode of action based on a toxic elevation in cytosolic Ca^2+^. Consistent with this, estragole and methyl eugenol both failed to evoke measurable Ca^2+^ elevation using a concentration (i.e. 3.2 mM) which is 2 to 3 times greater than the determined IC_50_. It is noteworthy that the IC_50_ values determined for the eugenol derivatives are unlikely to simply reflect differences in hydrophobicity because acetyl eugenol, isoeugenol and eugenol (which represent the full range of IC_50_ values observed in the present study) have virtually identical hydrophobicity (log P) values of approximately 2.5 [14].

In contrast to the present study, the amplitude and duration of cytosolic Ca^2+^ elevation in response to amiodarone and carvacrol correlates with drug toxicity [7], [8]. The fungicidal activity of amiodarone has been shown to be tightly coupled to Ca^2+^ influx across the plasma membrane; reducing extracellular Ca^2+^ with EGTA blocks cytosolic Ca^2+^ elevation and rescuses growth inhibition by amiodarone [7]. Furthermore, amiodarone toxicity is also dependent on hyperpolarisation of the yeast plasma membrane and consequently increases the driving force for Ca^2+^ influx [18]. Consistent with a cytotoxic influx of Ca^2+^ mediating amiodarone and carvacrol toxicity, *vma2Δ* mutants which lack vacuolar H^+^ pumping activity (and as a consequence have impaired Ca^2+^ sequestration into the vacuole via Ca^2+^/H^+^ antiport) exhibit prolonged cytosolic Ca^2+^ elevations and have increased sensitivity to amiodarone and carvacrol [19], [8]. We adopted a similar approach in order to investigate the role of the vacuole in sequestering Ca^2+^ from the cytosol and to negate eugenol toxicity using the *pmc1Δ* (vacuolar Ca^2+^ ATPase; Figure 2N) and *vcx1Δ* (vacuolar Ca^2+^/H^+^ antiporter; Figure 2O) mutants. Both *pmc1Δ* and *vcx1Δ* exhibited similar sensitivity to eugenol as the parental wild type strain (BY4742) indicating that a reduced capacity to remove Ca^2+^ from the cytosol across the vacuolar membrane did not affect eugenol toxicity. Taken together, these data are consistent with eugenol toxicity being independent of a general cytotoxic Ca^2+^ elevation.

### Tolerance to eugenol is dependent on extracellular Ca^2+^ influx via Cch1p

**Figure 3:**
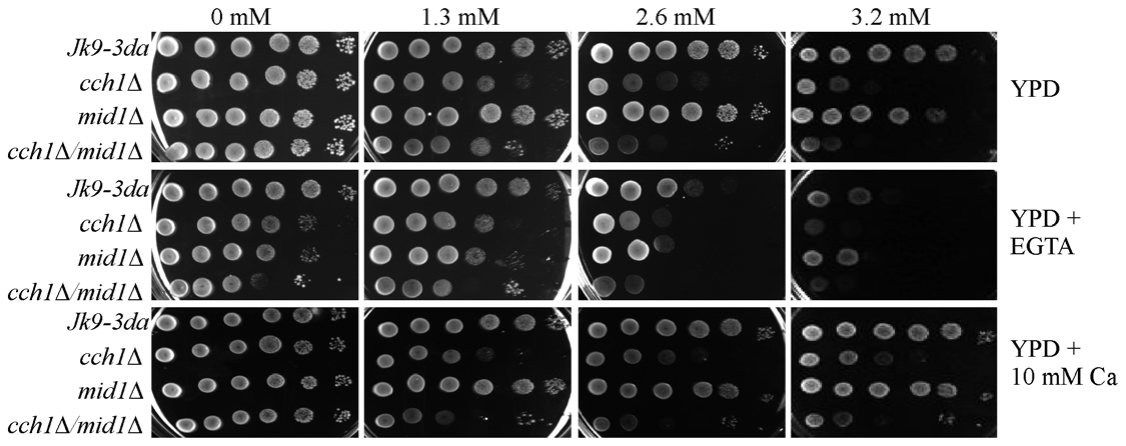
Extracellular Ca^2+^ is necessary for eugenol tolerance. Yeast cultures were spotted onto YPD, YPD supplemented with 10_2_ agar plates containing either 0 (1% ethanol), 1.3, 2.6 or 3.2 mM eugenol. Left-most spots on each plate are growth after 2 days at 30 C after inoculation with 5 µl culture at approximately 0.5×10^8^ cells/ml. Serial 10-fold serial dilution of the left-most inoculum is shown to the right.

Previous studies showed that Cch1p (but not Mid1p) was a crucial factor in determining the tolerance of *S. cerevisiae* to eugenol; the hyper-sensitivity of *cch1Δ* mutants suggested that a Cch1p-mediated Ca^2+^ influx may be necessary to protect yeast against the toxic effects of eugenol [9]. To investigate this possibility further, dot drop growth assays on YPD media supplemented with eugenol and either 10 mM CaCl_2_ or 10 mM EGTA (to increase or reduce extracellular Ca^2+^ respectively) were conducted. Reducing extracellular Ca^2+^ reduced the tolerance of the wild type (Jk9-3da) and *mid1Δ* strains to levels similar to that observed for the *cch1Δ* mutants (Figure 3). Furthermore, the sensitivity of the *cch1Δ* strains to eugenol was unaffected by the reduction of extracellular Ca^2+^ using EGTA. These data suggest that Cch1p-mediated Ca^2+^ influx is necessary for eugenol tolerance in yeast rather than a Ca^2+^ influx *per se*. Consistent with this, the eugenol sensitivity of the *cch1Δ* mutants could not be rescued by supplementing the growth media with additional extracellular Ca^2+^. Furthermore, the inability to enhance the tolerance of yeast growth to eugenol by increasing extracellular Ca^2+^ content of the growth media also indicates that there is sufficient Ca^2+^ in YPD (approximate Ca^2+^ content of 100–200 µM; [20]) to support Cch1p-mediated Ca^2+^ influx. This is consistent with previous reports which show that Cch1p mediates high affinity Ca^2+^ uptake in yeast (reviewed by [21]). Figure 3 is also consistent with the previous observation that Cch1p operates independently of Mid1p in response to eugenol stress [9]. Although it is generally accepted that Mid1p and Cch1p function together as a high affinity Ca^2+^ influx mechanism in S. cerevisiae (e.g. [10]) Cch1p activity independent of Mid1p has also been reported for Li stress at high temperature [22]. Finally, the observation that the *cch1Δ* yeast mutants are able to survive 10 minute exposure to eugenol at 3.2 mM (Figure S1, A & G) but not persistent exposure at 3.2 mM eugenol (Figure 3) indicates that the Cch1p-dependent tolerance mechanism operates to enhance the survival of yeast to persistent (long term) exposure to eugenol.

**Figure 4:**
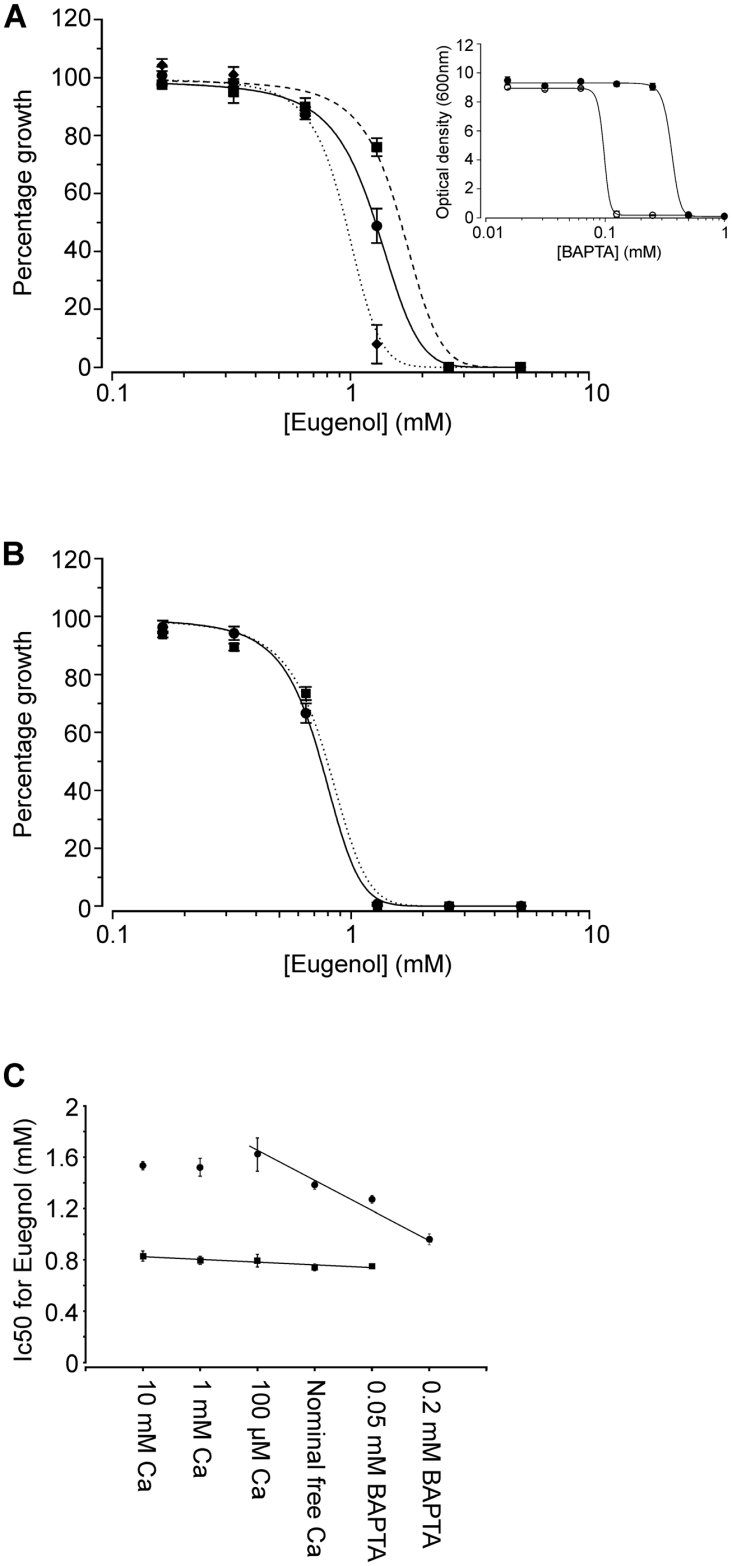
Dependence of eugenol tolerance on Cch1p-mediated Ca^2+^ influx. A) Dilution assays of eugenol activity versus *Jk9-3da* growth in SCM-leu-Ca supplemented with 100 µM CaCl_2_ (▪), 50 µM BAPTA (•) and 200 µM BAPTA (♦). Absorbance was recorded after 48 hours incubation and is shown as growth (absorbance) relative to growth exhibited by yeast in eugenol-free control media. Data are fitted with the dose-response function min + (max-min)/1+ ((x/IC50)^−p^)) where p is the slope, IC50 is the eugenol concentration inhibiting 50% growth and min and max represent minimum and maximum relative absorbance values respectively. Mean values (± SEM) from 4 experiments are shown. *Inset*: Dilution assays showing growth of *jk9-3da* (solid symbols) and *cch1Δ* (open symbols) cells in SCM-leu-Ca supplemented with BAPTA. Growth is shown as optical density (600 nm) after 48 hours and data are fitted with the dose-response function used in ‘A’. IC50 for BAPTA is 359±35.9 µM and 97.7±12.4 µM for *jk9-3da* and *cch1Δ* cells respectively. Mean values (± SD) from 3 experiments are shown. B) as ‘A’ except growth of *cch1Δ* strain in SCM-leu-Ca supplemented with 100 µM CaCl2 (▪), 50 µM BAPTA (•). C) IC50 for eugenol plotted as a function of CaCl_2_ and BAPTA added to SCM-leu-Ca growth media for *Jk9-3da* (•) and *cch1Δ* (▪) strains. IC50 values where obtain from fits of data as shown in parts ‘A’ and ‘B’. Data are fitted with linear regression fits for *Jk9-3da* (r^2^ = 0.8775) and *cch1Δ* (r^2^ = 0.8694).

In order to define the dependence of eugenol tolerance on extracellular Ca^2+^ more precisely, growth experiments were conducted using a Ca^2+^-free synthetic complete medim (SCM-leu-Ca); this permitted a more robust yet subtle control of extracellular Ca^2+^ levels using the Ca^2+^ chelator, BAPTA (which exhibits higher specificity for Ca^2+^ and is less pH sensitive than EGTA). Figure 4A shows that the IC_50_ of eugenol (the concentration of eugenol resulting in 50% inhibition of growth when compared to growth in the absence of eugenol) for the wild type strain was strongly dependent on extracellular Ca^2+^. In the presence of 100 µM extracellular Ca^2+^, the IC_50_ of eugenol for wild type yeast was 1.62±0.133 mM and this decreased to 1.27±0.031 and 0.96±0.043 mM in the presence of 0.05 and 0.2 mM BAPTA, respectively. Importantly, the growth of wild type yeast in the absence of eugenol was unaffected by the presence of 0.05 and 0.2 mM BAPTA (Figure 4A, inset).

In contrast to wild type yeast, the addition or removal of extracellular Ca^2+^ did not affect the sensitivity of the cch1*Δ* strain to eugenol (Figure 4B and C). The sensitivity of the cch1*Δ* mutant to eugenol (IC50 = 0.796±0.045 mM in the presence of 100 µM Ca^2+^) was similar to that for wild type yeast in the presence of 0.2 mM BAPTA (0.96±0.043 mM) indicating that the Ca^2+^-dependent tolerance to eugenol exhibited by wild type yeast is dependent on Ca^2+^ influx via Cch1p. Furthermore, the cch1*Δ* mutant was more sensitive to reductions in extracellular Ca^2+^ than wild type yeast as it was unable to grow in media containing more than 0.0625 mM BAPTA (Figure 4A inset). These data support the yeast growth patterns shown in Figure 3 and illustrate that in low Ca^2+^ conditions, Cch1p is required for Ca^2+^ homeostasis. In addition, they are also consistent with previous observations that Ca^2+^ uptake in cch1*Δ* mutants is approx. 5-fold less than wild type yeast under non-stress conditions [10] and with the widely accepted dogma that Cch1p forms part of the high affinity Ca^2+^ influx system (HACS; [23], [24]) which is essential to maintain growth in low Ca^2+^ environments.

Taken together, the data show that Ca^2+^ influx via Cch1p is necessary for yeast tolerance to eugenol. In addition, the observation that increasing extracellular Ca^2+^ to levels greater than 100 µM does not enhance the tolerance of wild type yeast to eugenol (Figure 2C) is in agreement with the dot drop experiments on YPD media (Figure 3) and is consistent with Cch1p-mediated Ca^2+^ transport being saturated at µM levels of Ca^2+^. Interestingly, similar studies conducted on *S. cerevisiae* have shown that the activity of antifungal azoles (e.g. miconazole) is also enhanced by extracellular Ca^2+^ sequestration (using 1 mM EGTA); however, in contrast to that observed with eugenol, the addition of 3 mM Ca^2+^ to the growth media reduced azole activity by 3-fold [13]. Furthermore, azole activity against yeast is enhanced in the presence of FK506 [13] suggesting a role for extracellular Ca^2+^ influx and calcineurin activation in the azole tolerance mechanisms employed by yeast. Interestingly, eugenol tolerance is independent of calcineurin activation [9] which serves to highlight differences between the Ca^2+^-dependent tolerance mechanisms employed by yeast in response to the antifungal properties of eugenol and the commercially available azoles.

In conclusion, our data show that a toxic elevation in cytosolic Ca^2+^ elevation is unlikely to be responsible for eugenol toxicity in yeast and that the role of Ca^2+^ in eugenol toxicity appears confined to a Cch1p-dependent Ca^2+^ influx which is necessary to enhance eugenol tolerance in yeast. Although the downstream targets of the resulting Ca^2+^ signal remain unknown, our data suggest that the signalling pathways employed by yeast to tolerate eugenol toxicity will be distinct to those employed in azole and amiodarone tolerance. This is supported further by the discovery that eugenol and amiodarone employ different modes of action with respect to antifungal activity. These differences highlight the potential to use eugenol in combination therapies which aim to augment the efficacy of commercially available azoles and other promising antifungal drugs.

## Supporting Information

Figure S1 **Viability of** ***cch1Δ*** **(A and G),** ***mid1Δ*** **(B and H) and** ***cch1Δmid1Δ*** **(C and I) cells after 10 minutes exposure to varying concentrations of eugenol suspended in Ca buffer (A, B, C) and BAPTA buffer (G, H, I).** Yeast cultures are spotted on to SCM-leu media containing 2% agar; left most spots are growth after 2 days following inoculation with 5 µl of culture. Serial 10-fold dilution of the left most inoculum is shown to the right. Ca^2+^-dependent aequorin luminescence from *cch1Δ* (D and J), *mid1Δ* (E and K) and *cch1Δmid1Δ* (F and L) cells in response to 3.2 and 9.6 mM eugenol in Ca buffer (D, E, F) and BAPTA buffer (J, K, L). Eugenol was added at 40 seconds (indicated by arrow). Traces represent mean (± SEM) from at least 5 independent experiments. SEM vales are illustrated using grey shading. Luminescence was recorded every 0.2 seconds and is expressed in arbitrary units (AU). Inset is data from the main figure on an expanded y axis.
(TIF)
