# Supplementary figures and images for: Calcium Dependence of Eugenol Tolerance and Toxicity in Saccharomyces cerevisiae
Source: PLoS One. 2014 Jul 18;9(7):e102712. doi: 10.1371/journal.pone.0102712 (PMC4103870; doi:10.1371/journal.pone.0102712)

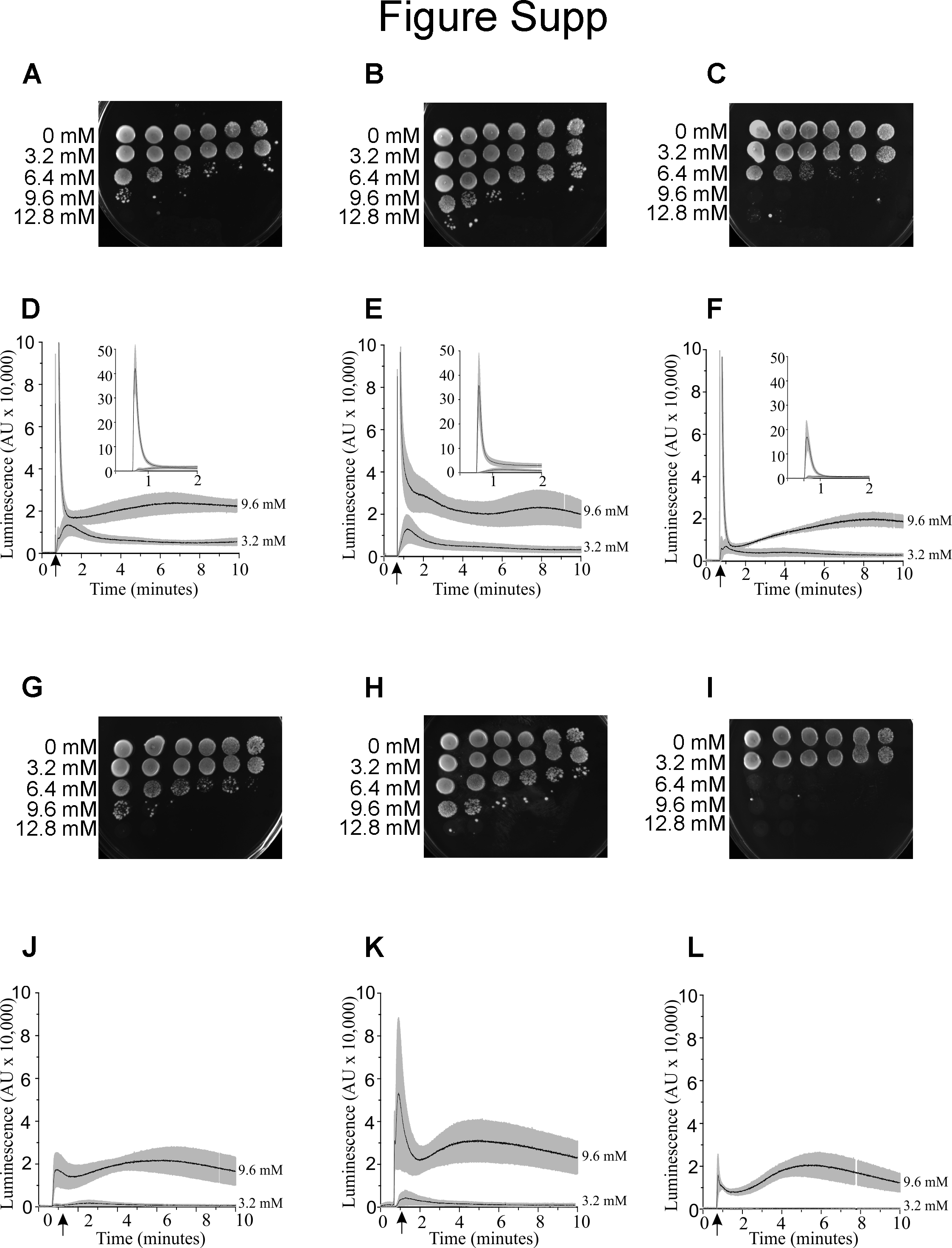

Supplement: Figure S1 — Viability of cch1Δ (A and G), mid1Δ (B and H) and cch1Δmid1Δ (C and I) cells after 10 minutes exposure to varying concentrations of eugenol suspended in Ca buffer (A, B, C) and BAPTA buffer (G, H, I). Yeast cultures are spotted on to SCM-leu media containing 2% agar; left most spots are growth after 2 days following inoculation with 5 µl of culture. Serial 10-fold dilution of the left most inoculum is shown to the right. Ca2+-dependent aequorin luminescence from cch1Δ (D and J), mid1Δ (E and K) and cch1Δmid1Δ (F and L) cells in response to 3.2 and 9.6 mM eugenol in Ca buffer (D, E, F) and BAPTA buffer (J, K, L). Eugenol was added at 40 seconds (indicated by arrow). Traces represent mean (± SEM) from at least 5 independent experiments. SEM vales are illustrated using grey shading. Luminescence was recorded every 0.2 seconds and is expressed in arbitrary units (AU). Inset is data from the main figure on an expanded y axis. (TIF) [file pone.0102712.s001.tif]
